# Can type 1 diabetes be an unexpected complication of obesity?

**DOI:** 10.3389/fendo.2023.1121303

**Published:** 2023-03-31

**Authors:** Paulina Oboza, Natalia Ogarek, Magdalena Olszanecka-Glinianowicz, Piotr Kocelak

**Affiliations:** ^1^ Students’ Scientific Society at the Pathophysiology Unit, Department of Pathophysiology, Faculty of Medical Sciences in Katowice, The Medical University of Silesia, Katowice, Poland; ^2^ Pathophysiology Unit, Department of Pathophysiology, Faculty of Medical Sciences in Katowice, The Medical University of Silesia, Katowice, Poland; ^3^ Health Promotion and Obesity Management Unit, Department of Pathophysiology, Faculty of Medical Sciences in Katowice, The Medical University of Silesia, Katowice, Poland

**Keywords:** type 1 diabetes, overweight, obesity, autoimmunity, β-cell destruction

## Abstract

Type 1 diabetes (T1D) is one of the most common chronic autoimmune diseases, characterized by absolute insulin deficiency caused *via* inflammatory destruction of the pancreatic β-cell. Genetic, epigenetic, and environmental factors play a role in the development of diseases. Almost ⅕ of cases involve people under the age of 20. In recent years, the incidence of both T1D and obesity has been increasing, especially among children, adolescents, and young people. In addition, according to the latest study, the prevalence of overweight or obesity in people with T1D has increased significantly. The risk factors of weight gain included using exogenous insulin, intensifying insulin therapy, fear of hypoglycemia and related decrease in physical activity, and psychological factors, such as emotional eating and binge eating. It has also been suggested that T1D may be a complication of obesity. The relationship between body size in childhood, increase in body mass index values in late adolescence and the development of T1D in young adulthood is considered. Moreover, the coexistence of T1D and T2D is increasingly observed, this situation is called double or hybrid diabetes. This is associated with an increased risk of the earlier development of dyslipidemia, cardiovascular diseases, cancer, and consequently a shortening of life. Thus, the purpose of this review was to summarize the relationships between overweight or obesity and T1D.

## 1 Introduction

It is estimated that type 1 diabetes (T1D) occurs in 8.4 million people worldwide, of which 1.5 million (18%) are aged below 20 years (1). The first peak of the disease occurs in children between 4 and 7 years and the second between 10 and 14 years (2). In recent years, the incidence of both T1D and obesity has increased. In addition, significantly increases the incidence of overweight and obesity among patients with T1D (3), most common among girls and patients with a diagnosis of T1D in puberty. As expected in obese children with T1D more often occurs common complications of obesity such as hypertension, dyslipidemia, and cardiac autonomic dysfunction (4). It has also been suggested that the development of obesity not only occurs in the course of T1D, but obesity in adolescents may also be a risk factor for developing T1D in early adulthood (5).

The incidence of T1D has increased markedly in the second half of the 20^th^ century (6). It has also been reported that the global incidence of T1D increases by 2.8% and in Europe by 3.9% annually (7). The International Diabetes Federation report from 2022 showed that 62% of new cases of T1D were diagnosed in subjects over the age of 20 (8). This suggests that environmental factors play an important role in the development of T1D (9, 10). Interestingly, the study from the Kuwait population showed a parallel increase in the incidence of T1D and obesity over the last almost 30 years (11). Thus, the purpose of this review was to summarize the relationships between overweight or obesity and T1D.

## 2 Pathogenesis of T1D

T1D is a chronic autoimmune disease characterized by absolute insulin deficiency related to the inflammatory destruction of the pancreatic β-cell. In the development of T1D genetic, epigenetic, and environmental factors play a role. The main genetic factors are mutations of the regions in human leukocyte antigens (HLA) class II on chromosome 6p21. Moreover, mutations of other loci from beyond the Major Histocompatibility Complex such as IFIH1, IL2RA, PTPN22, and CTLA4 are described. Subjects heterozygous for HLA-DRB1*04 and HLA-DRB1*03 types have the highest risk of developing T1D. Overexpression of the human leukocyte antigen or HLA class molecules DR4, DQ8, and DQ2 and action of one or more environmental factors cause misdiagnosis of β-cell components by the immune system and the development of inflammation (12).

The most common antibodies found in the serum of T1D patients are insulin autoantibodies(IAA), glutamic acid decarboxylase antibodies (GADA), islet antigen 2 antibodies (IA-2A),and zinc transporter 8 antibodies (ZnT8A). The antibodies are present in the blood long before the clinical symptoms of the disease. However, the main factor of β-cell damage is inflammation, mediated by CD8+ T cells, CD4+ T cells, macrophages, and B cells. In addition, all pancreatic islet cells over-express HLA class I, associated with the local production of interferons (13). This local production of interferons by β-cell initiates the recruitment of immune cells and the activation of the NF-κB pathway related to proapoptotic actions. In the inflammatory microenvironment of the pancreatic islets, there is a facilitated infiltration of naïve and non-islet-reactive T cells except for activated T cells, their influx is associated with increased vascular permeability. Moreover, cytokines induce the excessive production of reactive oxygen species and the activation of caspases, and these species activate proinflammatory pathways. Furthermore, beta-cells actively participate in enhancing pathogenic processes by compensatory mechanisms activated in response to immune stress and act as effective antigen-presenting cells. The hybrid and chimeric neoepitopes play a role in CD4+ T-cell activation (14). The main mechanism of β-cell death is apoptosis induced directly by contact of autoreactive T lymphocytes with β-cell *via* the perforating system or Fas/Fas ligand interaction (15).

## 3 Environmental risk factors of the development of T1D

The penetrance of main susceptibility genes or low-risk genes is affected by epigenetic and environmental factors and this interaction plays a key role in triggering autoimmunity. One of the well-known environmental factors that play a role in the development of T1D is viral infections including herpesviruses, rotaviruses, enteroviruses, rubella, and mumps viruses (16). It has also been suggested that the occurrence of viral respiratory tract infection in early life may favor the development of T1D many years later (17, 18). Recently, SARS-CoV-2 joined this group of viruses (19–22). On the other hand, in accordance with the hygiene hypothesis, some experimental studies suggested that some viral infections may play a protective role (16).

Numerous studies assessed the association between birth weight and the risk of the development of T1D but their results are inconclusive. The meta-analysis of 29 studies including 12,807 patients with T1D showed that greater birth weight is a significant risk factor for T1D development (23). Another meta-analysis involving 2,398,150 subjects including 7,491 with T1D also found positive correlations between higher birth weight and T1D risk (24). In contrast, Pacaud et al. (25) observed that the risk of autoimmunity related to the development of T1D is independent of birth weight but is higher in children of diabetic mothers with rapid weight gain during the first 2 years of life. For the first time, the link between rapid weight gain during infancy and the higher risk of the development of T1D was described more than 20 years ago in the Finnish population. However, in this study, an independent risk factor was also exposure to cow’s milk formula-feeding before 3 months of life (26). Besides birth weight and infancy or childhood weight gain, another factor that may contribute to the development of T1D is rapid linear growth (24, 27). It has been suggested that the increasing incidence of T1D in developed countries is the result of the growing prevalence of obesity among children and secular changes in linear growth (28). This potential mechanism is described in more detail in the fifth paragraph of the manuscript.

The next risk factor for the development of T1D is the microbiome (29). It has been shown that gut microbiota in T1D patients is distinctly different from healthy subjects, mainly with fewer bacteria essential to maintain gut integrity (30). Furthermore, decreased microbial diversity was described in both T1D patients (31) and children with autoantibodies (32). Moreover, it has been described that children delivered by cesarean section have a higher risk of the development of T1D associated with lack of contact with the mother’s vaginal microbiome hence, followed by abnormal colonization (33, 34).

## 4 The development of obesity in the course of T1D

The interaction between overweight/obesity and T1D seems to be bidirectional. Weight gain is a side effect of T1D treatment and is the risk factor for its development.

An international, prospective, multicenter SWEET study, diabetes registry including over 23,000 children with T1D showed the occurrence of overweight and obesity in 31.8% of them. The prevalence of obesity among younger children was significantly higher in males (9.6% vs. 6.2%, p < 0.0001) and among the oldest in females (7.8% vs. 6.2, p < 0.0001) (35).

Moreover, the Pittsburgh Epidemiology of Diabetes Complications Study found a faster increase in the incidence of obesity among people with T1D than in the general population during the observation from 1986 to 2007 (36). Similar data were obtained in the Epidemiology of Diabetes Interventions and Complications trial during 12 years of follow-up (37). In addition, a recently published study from the US showed the prevalence of obesity in 62% of patients with T1D and 64% of control subjects (38). Weight gain in patients with T1D is related to insulin therapy, especially intensive (IIT), and a tendency to low physical activity for fear of hypoglycemia as well as increased consumption of simple carbohydrates before or during exercise (39, 40).

Physiologically secreted insulin by the portal vein is transported to the liver from where 40-50% enters the systemic circulation. Whereas, exogenous insulin administered subcutaneously bypasses the portal vein circulation. Thus, exogenous insulin has a greater effect on adipose tissue and muscle than on the liver, which promotes the accumulation of adipose tissue during the positive energy balance. Hyperinsulinemia can also increase hunger and food intake (41). All of its actions are particularly intensified during the IIT (42, 43).

Moreover, it has been shown that the fear of hypoglycaemia significantly reduces physical activity in both children/adolescents and adults with T1D (44). One study found that adults with a recently diagnosed T1D spent on average a quarter less time in moderate-to-vigorous physical activity per day than healthy adults (45). The role of psychological factors cannot be overlooked either. Disease-related poorer quality of life, difficulties in maintaining normal body weight, ideal body image promoted by media, body dissatisfaction, and fear of hypoglycaemia may be causes of the development of depression and emotional eating (3). In addition, an increased risk of weight gain is associated with unhealthy habits and altered eating behaviours. A sedentary lifestyle and the availability of energy-dense diets are a cause of positive energy balance and the development of obesity. Disturbed β-cell-secreted regulators of food intake such as insulin and amylin may result in changes in food intake and promote weight gain. Moreover, pancreatic alpha-cells dysfunction results in deficient glucagon secretion in hypoglycaemia and dysregulation of postprandial glucagon secretion (40). The additional risk factors included female sex, T1D duration, older age, ethnicity (Hispanic/Latino race), lower education level, disease onset during puberty, or low weight at T1D onset (46).

Treatment of obesity in T1D is essential for both better glycemic control and preventing the development of diabetes-related complications. It involves a multidisciplinary approach that includes lifestyle and behavioural interventions (ie, dietary modification, and physical activity). Although physical activity improves insulin sensitivity, it may be associated with an increased risk of hypoglycaemia in T1D patients, which can be reduced by advances in hybrid, closed-loop, artificial pancreas systems. With regard to dietary modification, to date, there is a paucity of comprehensive studies on what diet would be most beneficial in the T1D population. Furthermore, promising effects on weight control have been reported with the use of pharmacological agents as adjunctive therapies to reduce insulin dosage. Benefits have been reported with metformin (by improving insulin sensitivity), pramlintide (by delaying gastric emptying and suppressing glucagon and appetite), GLP1 receptor agonists (by incretin-based effects) and SGLT inhibitors (by glucosuria). The registered adjunctive therapy for long-term use in people with T1D in the USA is pramlintide, while in Europe and Japan, SGLT inhibitors have been approved for T1D with overweight or obesity. Finally, a final therapeutic option could be bariatric surgery in T1D patients in whom there are no results through lifestyle modifications or adjunctive therapies. However, only a handful of studies have been published, limited by small sample sizes and short-term results (39).

**Figure 1:**
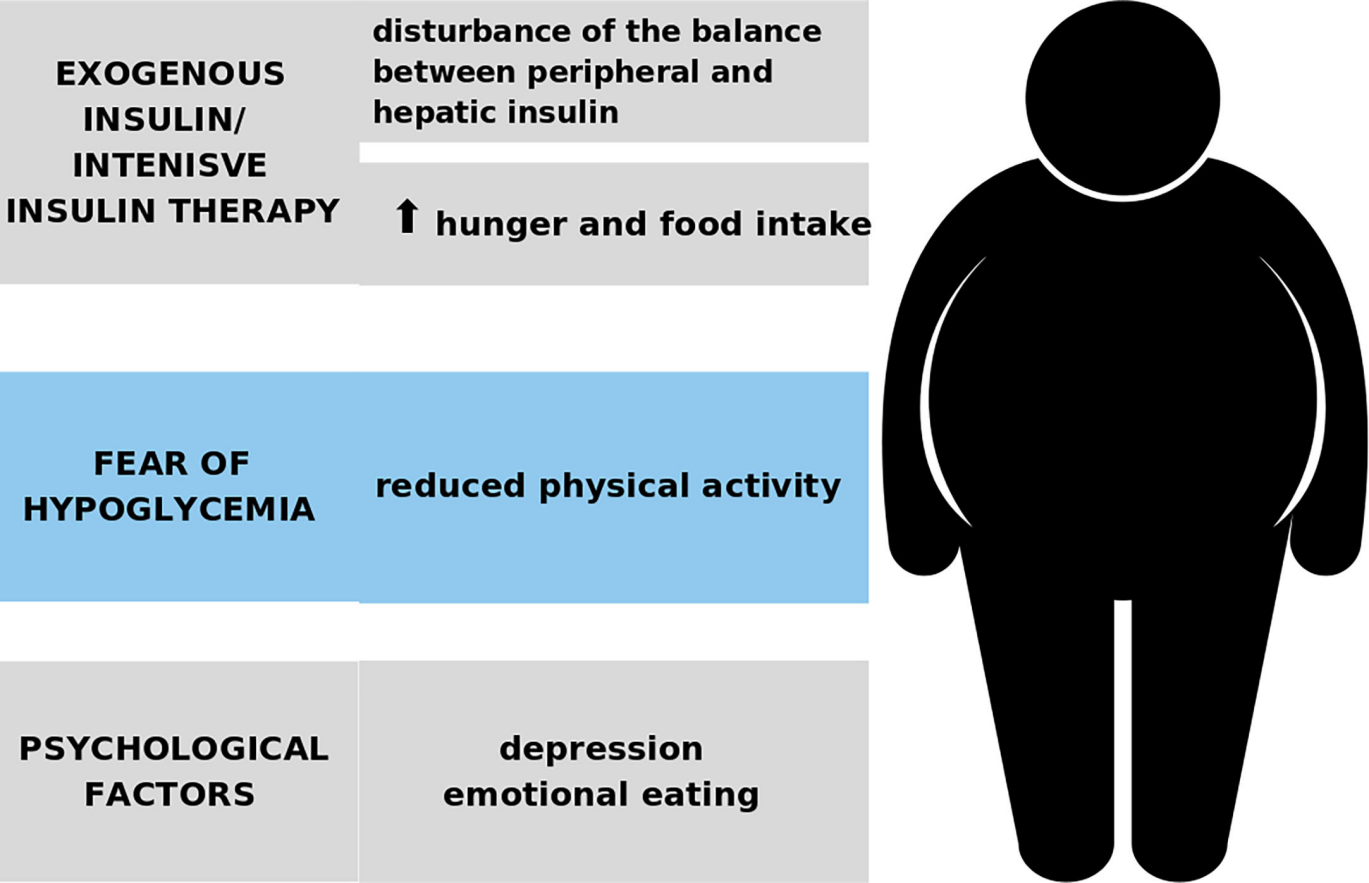
The development of obesity in the course of T1D.

The mechanism of the development of obesity in T1D is shown in **Figure 1**.

## 5 Mechanism of T1D development in patients with overweight or obesity

Type 2 diabetes (T2D) is one of the most common complications of overweight and obesity. Successful treatment of obesity prevents the development of T2D and can also cause its remission (47). Although there is little evidence that overweight and obesity are involved in the pathogenesis of the development of T1D, several mechanisms may be considered to explain the link between obesity and the increased incidence of T1D. Another possible mechanism is the impact of excess adipose tissue on the function of the immune system (39). Chronic systemic inflammation related to obesity plays a mediatory role in the deregulation of Th17/Treg balance and increased number of Th1 cells (48). Another obesity-related immunologic cell alteration takes place locally in adipose tissue and comprises lower B-regulatory and invariant NK cell numbers within the adipose tissue that results in inflammatory cell infiltration, decreased insulin sensitivity, and dysregulation of adipokine secretion (49). Increased lipolysis related to local inflammation, hormonal disturbances, and insulin resistance results in the release of more free fatty acids (FFA). FFA promotes pro-inflammatory M1-macrophage infiltration and activates NOD-like receptor protein 3 (NLRP3)-inflammasome, resulting in IL-1β and IL-18 secretion. Both these interleukins are involved in the pathogenesis of autoimmune diseases (50). Further, adipocytes undergo apoptosis and release antigens that were previously unavailable for the immune system (51). In addition, apoptosis inhibitors of macrophages stimulate the production of IgG autoantibodies *via* immune complexes with natural autoreactive IgM associated with autoantigens forms (50). This hypothesis is supported by an observational study that showed significantly higher circulating proinflammatory adipokines and cytokines levels in obese than normal-weight children with new onset of T1D (52). Furthermore, the TrialNet Pathway to Prevention Study showed a higher risk of islet autoantibodies expression and developing T1D in relatives with overweight and obesity of patients with T1D, independently from the occurrence of T1D–related HLA alleles (53, 54). Moreover, Buzzetii et al. (55) showed that the frequency of occurrence of islet antigen-2 autoantibodies (IA-2)_256-760_ increased with BMI values in subjects with adult autoimmune latent diabetes, suggesting a possible different pathogenetic mechanism.

In 2001, Wilkin published a provocative and controversial hypothesis, named the “Accelerator Hypothesis”, suggesting that the divergence between T1D and T2D blurs and considers those two are the same disorder of insulin resistance set against the different genetic backgrounds. Both these diseases are distinguishable only by the rate of β-cell loss and the “accelerators” such as insulin resistance, β-cell autoimmunity, and genetic background. Regardless of the type of diabetes, finally comes with total insulin dependence (56). This hypothesis also suggests that increased demand for insulin related to insulin resistance may cause the accumulation of misfolded proteins in insulin-producing cells, and in consequence, endoplasmic reticulum stress, inflammation, the influx of immune cells, and the triggering of neoantigen exposure (57, 58).

Another hypothesis indicates that a diet with high fat, salt, and simple carbohydrate content, induces metabolic stress that contributes to early life β-cell fragility and increased susceptibility to T1D (59). The experimental studies conducted on a transgenic mice model indicated that T1D may be caused by immune-independent β-cell fragility and high-fat diet can stimulate genetic susceptibility (48, 60). Another study based on transgenic obese mice models with a mutation in a pleiotropic protein prohibitin (PHB) showed the development of obesity in both sexes regardless of the type of diet and the development of T1D-like phenotype in males on a high-fat diet only, related to mononuclear cell infiltration in the pancreas, and insulitis (61).

Additionally, dysbiosis related to high fat and simple carbohydrates low fibre diet may be a link between diet, obesity, and T1D (62–64).

**Figure 2:**
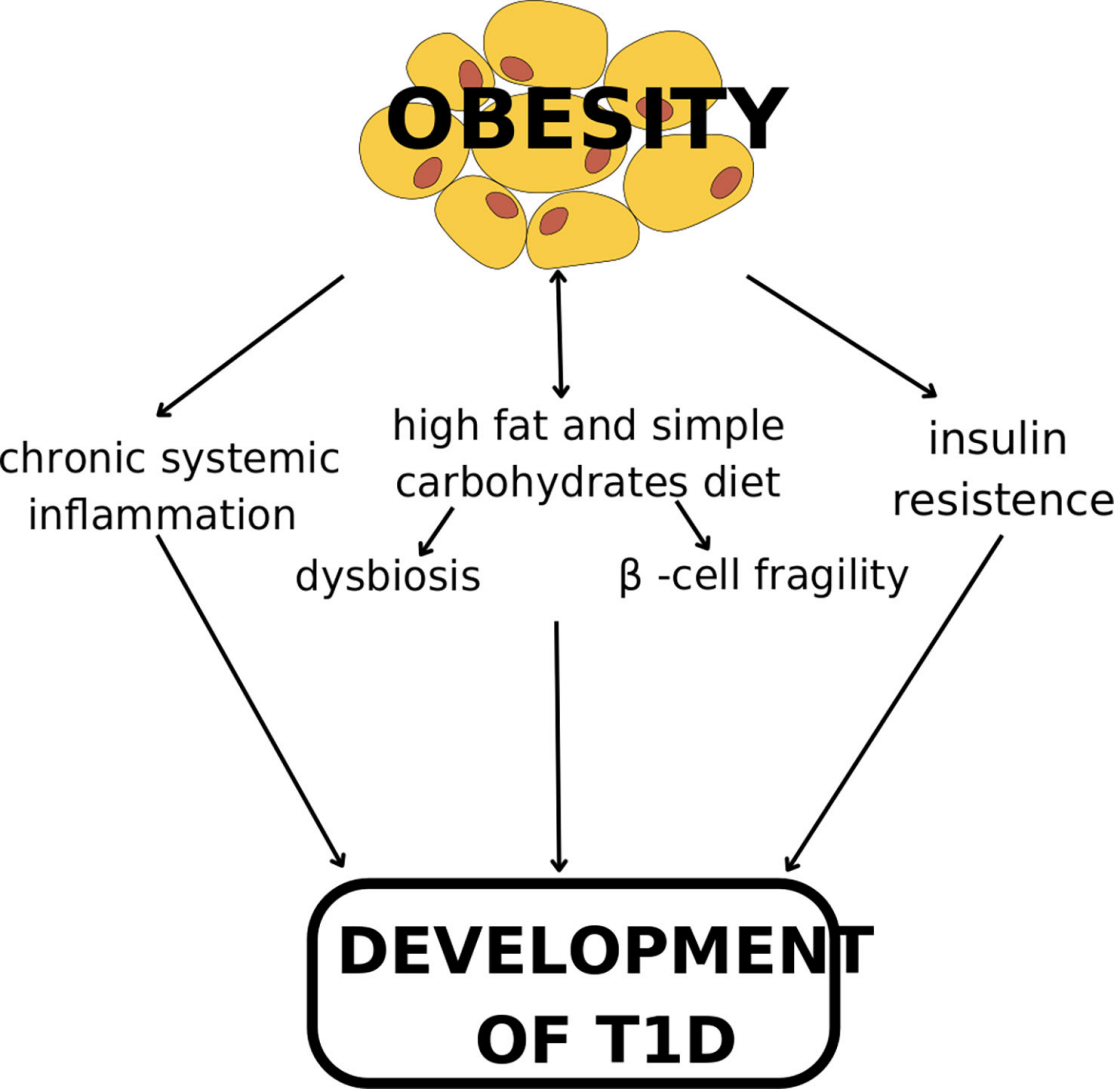
The mechanism of the development of T1D in overweight and obesity.

The potential mechanisms of the development of T1D in overweight and obesity are summarized in **Figure 2**.

## 6 The associations between body weight and the occurrence of T1D

The study using Mendelian randomization based on data from a large-scale T1D meta-analysis including 15,573 cases and 158,408 controls provided evidence that childhood body size at age of 10 years affects the development of T1D. This study shown also that childhood body size influences the risk for the development of asthma, eczema, and hypothyroidism although these effects are mediated by body size in later life. Thus, obesity in childhood is the risk factor for the development of T1D, while its effect on other immune-associated diseases is explained by the long-term effect of remaining overweight for many years over the life course (65). In addition, the study included Israeli military recruits’ incidence of T1D was 4.9 cases/per 100,000 person-years (777 incident cases) and increased across underweight and obesity from 3.6 to 8.4 cases per 100,000 person-years. Moreover, a 5-unit increase in BMI was associated a with 35% increase in the risk of T1D. Furthermore, the analysis of the association between adolescents’ BMI and T1D including the detection of at least one islet autoantibody, showed a gradual increase in the incidence of T1D across BMI categories from 2.38 cases per 100,000 person-years in normal BMI to 6.27 among obesity (5). In addition, the meta-analysis of four studies confirms the association between the subsequent development of T1D in children with obesity (OR 2.03, 95% CI 1.46-2.80). Moreover, a continuous relationship between childhood BMI and subsequent T1D was reported (OR 1.25, 95% CI 1.04-1.51) (66). Furthermore, the data from the Finish diabetic children population found that obesity after 3 years of age increased the risk of T1D more than twofold (28).

Another study including 5,913 patients with T1D and 8,823 without showed that in genetically predisposed children increase BMI by 1 standard deviation is associated with a 32% increased risk of the development of T1D (OR=1.32, 95%; CI 1.06–1.64) (67). In addition, the TRIGR study including over 2,000 children with a first-degree relative with T1D and increased HLA-conferred risk, during 10-14 years of follow-up showed that excessive weight at 2-10 years of life was associated with a twofold increase in the development of T1D (68). Of interest, it has been found that increased maternal BMI in the first trimester of pregnancy predisposes the child of parents without diabetes to T1D (69).

## 7 Double diabetes and diabetes complications

The latest classification replaced the terms ‘insulin-dependent’ and ‘non-insulin-dependent’ diabetes, reflecting disease pathogenesis with the terms T1D and T2D. However, as was described above there is an increasing overlap of these types of diabetes, which is why the concept of double diabetes or hybrid diabetes appeared at the beginning of this century (70).

Moreover, Josefsen et al. (71) showed that the pathogenesis of T1D and T2D may share common pathophysiological links. It was found that at the time of T1D diagnosis, 30% of the beta-cells are preserved but inactive (72). The analysis of gene expression in the Langerhans islets revealed that genes related to ceramide and sphingomyelin synthesis and degradation, secretion, circadian rhythm and insulin action, as well as changes resembling fetal dedifferentiation and asynchrony, previously described for T2D characteristics may also constitute mechanisms underlying beta-cell inactivity in T1D (71).

It can be expected that in the coming years, this may become the reason for the increasing frequency of complications of obesity and diabetes in patients at a younger age. In the development of the complications, an important role plays insulin resistance related to obesity and participated in the development of T2D, and hyperinsulinemia related to insulin therapy for T1D. Dyslipidemia and hypertension related to insulin resistance, microinflammation, and adipokines release disturbances occurring in children with obesity and new-onset T1D increased the risk of the early development of atherosclerosis and cardiovascular diseases (9, 53, 73, 74). It should also be noted that in children with obesity and T1D lipid profile is comparable to occurring in patients with T2D (75).

Moreover, obesity in patients with T1D increased the risk of the development of heart failure (HF) and HF-related death (76). Thus, in terms of macrovascular complications, obesity is T2D equivalent. Moreover, obesity is a risk factor for the development of microvascular complications in patients with T1D. It has been shown that obesity increased the risk of the development of diabetic retinopathy, independently from sex and glycemic control (OR=1.08; CI 1.04-1.16 per unit (kg/m^2^) increase in BMI) (77). A significantly higher risk of the progression of diabetic retinopathy has also been found in overweight and obese children with T1D (78). In addition, data from the National Diabetes Audit showed that obesity increased the risk of the development of diabetic kidney disease (DKD) in patients with T1D (79). Interestingly, a higher occurrence of DKD was found in patients with obesity and T1D than in T2D (80).

It should also be noted that the coexistence of obesity and T1D as well as obesity and double diabetes is a crucial risk factor for the development of many cancers. Insulin resistance combined with the use of exogenous insulin causes the activation of the mitogen-activated protein kinase (MAPK) pathway. A higher incidence of cancers such as colorectal, kidney, prostate, breast, ovarian, endometrial, pancreatic, oesophagal (81), thyroid, gallbladder, liver, leukemia, multiple myeloma, and non-Hodgkin lymphoma were reported (82, 83). The analysis of the Australian national diabetes registry including 953,382 patients registered between 1997 and 2008 linked to national death and cancer registries showed a higher standardized incidence of the pancreas, liver, esophagus, colon, stomach, thyroid, lung, endometrial, and ovarian cancers in patients with T1D. While among patients with T2D, a higher standardized incidence of almost all site-specific cancers was found with the highest value for liver and pancreas cancers (84). In addition, a five-country study of 9,000 cancers in patients with T1D showed a 15% higher risk of cancer in men and 17% in women with T1D (85). While the analysis of the data of 428,326 patients with T2D from the Finnish National Diabetes Register linked with the Finnish Cancer Registry between 1988 and 2014 years found a 16% higher cancer risk in diabetic patients than in the general population (86). However, it should be noted that none of the studies assessing the risk of cancer in diabetic patients considered the role of obesity. Therefore, further studies are needed to clarify whether obesity is the main risk factor for the development of cancer and whether the presence of diabetes itself or the use of exogenous insulin contributes to the development of cancer.

## 8 In summary

There is increasing evidence that T1D may be a complication of obesity. The growing occurrence of obesity among children and adolescents raises concerns that in the near future, the number of cases of not only T2D but also T1D will increase. It can be expected that many young adults will be diagnosed with double diabetes, which will contribute to the earlier occurrence not only of cardiovascular diseases, but also of cancers, and consequently shortening of life. Therefore, global action is needed to effectively prevent and treat obesity in children and adolescents. In addition, it should be remembered that children, adolescents, and young adults with obesity should be diagnosed not only for T2D but also for T1D.

## Author contributions

PO and NO analysed the literature, and drafted the manuscript. MO-G and PK contributed to the manuscript concept and critically revised the manuscript. All authors have read and agreed to the published version of the manuscript. All authors contributed to the article and approved the submitted version. 
